# Rapid radiation in spiny lobsters (*Palinurus *spp) as revealed by classic and ABC methods using mtDNA and microsatellite data

**DOI:** 10.1186/1471-2148-9-263

**Published:** 2009-11-09

**Authors:** Ferran Palero, Joao Lopes, Pere Abelló, Enrique Macpherson, Marta Pascual, Mark A Beaumont

**Affiliations:** 1 Departament de Genètica, Facultat de Biologia, Universitat de Barcelona, Av. Diagonal 645, 08028 Barcelona, Spain; 2 Institut de Ciències del Mar (CSIC), Passeig Marítim de la Barceloneta 37-49, 08003 Barcelona, Spain; 3 Centre d'Estudis Avançats de Blanes (CSIC), Carrer d'Accés a la Cala Sant Francesc 14, 17300 Blanes, Spain; 4 School of Biological Sciences, University of Reading, Whiteknights, P.O. Box 228, Reading RG6 6AJ, UK

## Abstract

**Background:**

Molecular tools may help to uncover closely related and still diverging species from a wide variety of taxa and provide insight into the mechanisms, pace and geography of marine speciation. There is a certain controversy on the phylogeography and speciation modes of species-groups with an Eastern Atlantic-Western Indian Ocean distribution, with previous studies suggesting that older events (Miocene) and/or more recent (Pleistocene) oceanographic processes could have influenced the phylogeny of marine taxa. The spiny lobster genus *Palinurus *allows for testing among speciation hypotheses, since it has a particular distribution with two groups of three species each in the Northeastern Atlantic (*P. elephas, P. mauritanicus *and *P. charlestoni*) and Southeastern Atlantic and Southwestern Indian Oceans (*P. gilchristi, P. delagoae *and *P. barbarae*). In the present study, we obtain a more complete understanding of the phylogenetic relationships among these species through a combined dataset with both nuclear and mitochondrial markers, by testing alternative hypotheses on both the mutation rate and tree topology under the recently developed approximate Bayesian computation (ABC) methods.

**Results:**

Our analyses support a North-to-South speciation pattern in *Palinurus *with all the South-African species forming a monophyletic clade nested within the Northern Hemisphere species. Coalescent-based ABC methods allowed us to reject the previously proposed hypothesis of a Middle Miocene speciation event related with the closure of the Tethyan Seaway. Instead, divergence times obtained for *Palinurus *species using the combined mtDNA-microsatellite dataset and standard mutation rates for mtDNA agree with known glaciation-related processes occurring during the last 2 my.

**Conclusion:**

The *Palinurus *speciation pattern is a typical example of a series of rapid speciation events occurring within a group, with very short branches separating different species. Our results support the hypothesis that recent climate change-related oceanographic processes have influenced the phylogeny of marine taxa, with most *Palinurus *species originating during the last two million years. The present study highlights the value of new coalescent-based statistical methods such as ABC for testing different speciation hypotheses using molecular data.

## Background

The high dispersal potential of planktonic larvae usually results in genetic homogeneity over large distances in marine species, unless local adaptation or oceanographic barriers counteract this dispersal [1]. Because of such dispersal potential, ranges of marine organisms have frequently been considered to be vast, even though marine species can also exhibit cryptic speciation and fine-scale endemism [2-4]. Allopatric speciation in marine organisms is mainly thought of a vicariance process, where a species' geographic range becomes fragmented following changes in oceanographic conditions or disconnection of populations by lower sea levels, with a consequent divergence due to genetic drift [5]. However, allopatric speciation could also result from a founder effect, where a new population is established by a small number of individuals, often by long-distance dispersal, with subsequent restricted gene flow leading to speciation [6,7].

Spiny lobsters (Palinuridae Latreille, 1802) are one of the most commercially significant groups of decapod crustaceans and they are considered key predators in a variety of habitats [8-10]. The most striking feature of these lobsters is their flat-bodied crystalline larval phase, the phyllosoma larva, which is specially adapted for dispersal in oceanic waters [11,12]. The phyllosoma larva has a long planktonic life (up to 24 months) before metamorphosing into the puerulus stage, which is the transitional stage to a benthic existence [13,14]. Since it is generally assumed that such a long planktonic larval duration should promote high levels of gene flow and effectively counterbalance the speciation process [15,16], life history traits make spiny lobsters a suitable model for better understanding the speciation process in marine organisms with large dispersal capabilities.

**Figure 1:**
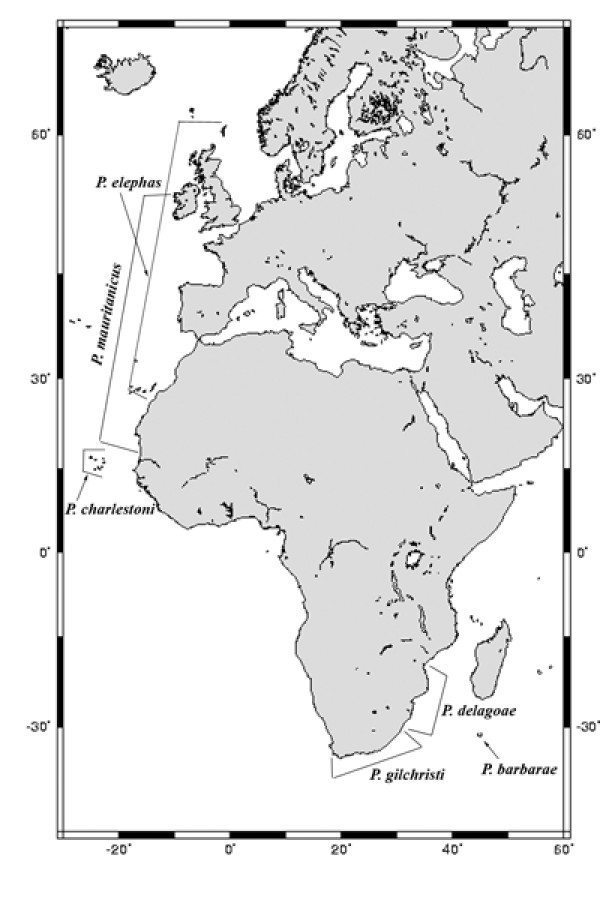
**The geographic distribution of Palinurus species**. Three species are present in the Northeastern Atlantic (*P. elephas*, *P. mauritanicus *and *P. charlestoni*) and other three in the Southeastern Atlantic and Southwestern Indian Oceans (*P. gilchristi*, *P. delagoae *and *P. barbarae*).

The genus *Palinurus*, a typically temperate-water genus within the Palinuridae [17], is particularly indicated for such studies since it has a well-defined distribution with two groups of three species each present in the Mediterranean and Northeastern Atlantic (*P. elephas *Fabricius, 1787, *P. mauritanicus *Gruvel, 1911, and *P. charlestoni *Forest and Postel, 1964) and Southeastern Atlantic and Southwestern Indian Oceans (*P. gilchristi *Stebbing, 1900, *P. delagoae *Barnard, 1926 and *P. barbarae *Groeneveld et al. 2006) (Figure 1). The phylogeny of *Palinurus *species has been recently addressed using Parsimony, Maximum Likelihood and Bayesian phylogenetic reconstruction methods on 16S and COI mtDNA sequences [18]. A strong statistical support was found for the monophyly of each species within the genus, but the interspecific relationships were poorly supported. Furthermore, Parsimony and Maximum Likelihood analyses were congruent (in some instances) while the Bayesian analyses failed to support any interspecific clustering, except for a *P. delagoae*/*P. barbarae *clade. Even though results were inconclusive, it was pointed out that the Northern Hemisphere species *P. charlestoni *could have been originated from a South-African ancestor colonizing Cape Verde islands. Therefore, it has been proposed that the present geographical distribution of *Palinurus *species indicates a pre-Miocene allopatric divergence, with two main lineages separating due to the closure of the marine gateway between Mediterranean Sea and Indian Ocean after the northward collision of Africa with Eurasia (11.2-23 Mya) [19].

Such interpretation implies that *Palinurus *mtDNA (COI and 16S rRNA combined) has evolved no faster than 0.18% (lower bound) to 0.36% (upper bound) per lineage per million years. Those rates of evolution would be 3-7 times slower than reported for other decapod taxa [20], with *Palinurus *showing among the slowest mtDNA mutation rate reported to date [18]. In relation to this question, there is a certain controversy on the phylogeography and speciation modes of Eastern Atlantic and Western Indian Ocean species of several taxa, such as algae [21], sea urchins [22] or fishes [23]. These studies suggest that older events during the Miocene (e.g. the closure of the Tethys Seaway) and/or recent oceanographic processes during the Pleistocene (e.g. Western Africa upwellings) could have influenced the phylogeny of marine taxa [24,25]. For example, an extensive review of antitropical biotas has shown that transequatorial migration is the most likely explanation for the origin of antitropic distributions of most near-shore marine taxa [26]. A more complete understanding of the phylogenetic relationships among *Palinurus *species will allow us to answer important questions regarding the speciation processes in marine taxa with an Eastern Atlantic and Western Indian Ocean distribution.

The advantage of constructing phylogenetic trees from genetic data has long been recognised, with the most widely used software in the field being MrBayes [27]. Moreover, it has more recently been appreciated that population genetic processes can lead to incongruence between the species tree and the genetic tree [28], for which new software considering separately the likelihood of the data given the gene tree and the likelihood of the gene tree given the species tree has been developed on top of MrBayes (BEST [29,30]). This software allows for better estimation of species trees from population genetic data, but the program can be applied strictly to sequence data. Since microsatellite markers allow for simultaneously sampling different genome regions, we opted to use a novel package that can deal with both sequence data and microsatellites jointly through the use of an approximate Bayesian computation (ABC) method [31].

**Figure 2:**
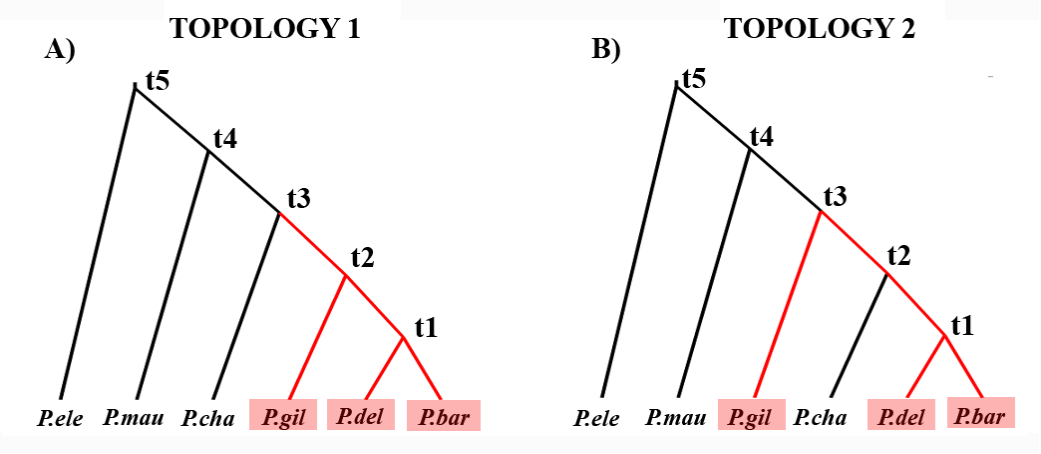
**Phylogenetic trees showing the alternative hypotheses tested in the present study**. The North-to-South speciation model (topology 1, with *P. charlestoni *originating from a *P. mauritanicus*-like ancestor) and the South-to-North speciation model (topology 2, with *P. charlestoni *originating from a south-african ancestor).

Therefore, the present study aims to ascertain phylogenetic relationships and monophyly patterns in species of the genus *Palinurus *from Eastern Atlantic and Western Indian Ocean using mitochondrial DNA sequence data and a set of 13 microsatellite markers. In order to solve the phylogenetic tree topology and test among opposite evolutionary hypotheses (Figure 2), we will use both classic distance-based and recently developed coalescent-based approximate Bayesian computation methods, which have been successfully used to trace complex colonizing scenarios [32]. Coalescent-based methods will allow us to define the likelihood for different mutation rates and tree topologies to have produced the observed dataset and consequently test the importance of an older mechanism (i.e. closure of marine gateway) versus more recent oceanographic processes in the origin of *Palinurus *species.

## Methods

### Taxon sampling and genotyping

DNA was obtained from *P. elephas *(n = 331; [33]), *P. mauritanicus *(n = 17; Atlantic Morocco; [33]), *P. charlestoni *(n = 5; Cape Verde Islands; [34]), *P. gilchristi *(n = 20; Southcoast South Africa; [35]), *P. delagoae *(n = 20; S-KZN2 South Africa; [36]) and *P. barbarae *(n = 18; Madagascar Ridge: Walters Shoals; [36]). Multiplex PCR amplification using the polymorphic microsatellite loci isolated from *P. elephas *was carried out under conditions described in Palero and Pascual [37]. Amplified products were scored using an ABI 3770 automatic sequencer from the University of Barcelona Scientific and Technical Services. Alleles were sized by PeakScanner™ software, with an internal size marker CST Rox 70-500 (BioVentures Inc.). The 16S rRNA and COI sequences were obtained as in [18] and Palero et al. [33,38]. All samples and microsatellite markers included in Palero [34] were used for classic distance-based analysis, while the combined dataset including mtDNA sequence data and 10 microsatellite loci (after excluding Pael22, Pael31 and Pael44 since they do not follow a "stepwise" change in allele size) was used for coalescent-based ABC analysis.

### Classic distance-based methods

We employed CONVERT 1.2 to transform the excel-based microsatellites dataset into different formats to be run by other population genetic programs [39]. Genetic diversity and pairwise differentiation estimates (F_ST_) for microsatellite data were obtained using the GENEPOP package version 4.0.7 [40], while average GTR corrected sequence divergence was obtained for mtDNA data as in [18]. Since different genetic markers can provide conflicting phylogenetic signals, a Mantel test was carried out correlating the genetic distance matrix obtained from mtDNA sequence data and the Fst distance matrix based on microsatellites. This will allow us to check for congruence among markers and for the possibility of combining them in a joint dataset. Isolation by distance patterns were evaluated by correlating geographic distance and each genetic distance through the Mantel test. If *Palinurus *speciation had occurred following a gradual North-to-South pattern, we would expect species most closely located geographically to be those separated by a smaller genetic distance, whereas we would expect a vicariance event and posterior contact between phylogenetically distant species (topology 2) to reduce the correlation between genetic and geographic distance matrices. The rows and columns of one of the matrices were subjected to 700 random permutations, with correlation being recalculated after each permutation. The Mantel test analysis for a multiple set of distance matrices has been implemented in R and is available from the authors upon request. Finally, the distance measure of Cavalli-Sforza and Edwards [41] was obtained from the combined mtDNA-microsatellite dataset by coding mtDNA haplotypes as individual alleles, and phylogenetic trees (based on individuals or species) were built using the Neighbor Joining algorithm as implemented in Populations v1.2.30 . The distance-based method has been shown to be among the most robust methods for phylogenetic reconstruction using microsatellite data [42-44]. A total of 1000 bootstrap replicates over loci were obtained to asses support for each clade. Previous phylogenetic analyses indicate that the species found in the Northernmost part of the distribution area of the genus, *Palinurus elephas*, is the most basal species of the group and therefore it has been used to root the trees [18,38].

### Approximate Bayesian computation implementation

When inferring phylogenies under the "Isolation with migration" model [45], likelihoods can only be computed for relatively simple scenarios containing few parameters [46]. Indeed, the likelihood function for complex demographic scenarios can be very difficult, and practically impossible, to solve analytically [47]. For this reason, application of ABC methods to solve phylogenetic inference-related problems has become of great interest [48-50]. ABC methods have the advantage of facilitating the comparison of alternative models marginal to parameter values without the need for calculating likelihoods [51]. The method relies on the simulation of large numbers of data sets using known parameters under a given coalescent model, for which it is more realistic than standard sequence-based phylogenetic approaches [30,52].

When dealing with coalescent-based inference, we rely on simulating genetic data based on a coalescence model and computing summary statistics from simulated datasets. A typical ABC approach involves two steps [51]: a rejection step and a regression adjustment and weighting step. The rejection step consists of accepting only the simulations whose summary statistics are close to the summary statistics obtained from the observed dataset. To assess this closeness, a Euclidian distance is computed between the entire set of normalized summary statistics and the normalized summary statistics calculated from the data. A set of parameter values is accepted when its Euclidian distance is within a certain percentage of the closest points to the studied data (as in [53]). The second step is a local linear regression adjustment that attempts to model the relationship between the parameter values and the summary statistics. This linear regression is performed only for the accepted set of parameter values. We assume that the relation between parameters and summary statistics is close to linear in the proximity of the target summary statistics. By using this adjustment, more points can be accepted, which allows a better characterization of the space problem [52]. Also in this step, each accepted set of parameter values is given a weight between zero and one that declines quadratically until a defined distance from the studied data set is reached (as in [48]).

To reduce heteroscedasticity in the regression, all demographic parameter values were transformed on a log scale. The transformed parameter values were adjusted one at a time using a general linear regression on the accepted points. Adjusted values were then back-transformed taking the exponential for all parameters, in order to present posterior densities on a normal scale [51,52]. The transformation also minimizes the appearance of values outside the prior ranges after performing the linear-regression correction. Previous studies indicated that the logistic and related transformations can lead to biases in the posterior densities estimated in the proximity of the prior boundaries under particular circumstances [31]. To avoid this problem we choose a log transformation, which still allows for points at the lower boundary to be retained within the support of the model. In this case, the points that fell outside the upper boundary after regression were discarded, since this procedure has been shown to give a more efficient estimation [31].

A standard backward coalescent process was implemented to simulate genetic data [54,55]. Simulated data are obtained by adding mutations under a stepwise mutation model [56] for short tandem repeats (STRs) and an infinite sites model [57] for sequence data. Hamilton and co-workers [58] suggest running several hundreds of thousands to millions of simulations, depending on the complexity of the underlying model. In our simulations 1,000,000 values of the summary statistics sets were generated and a tolerance δ = 0.01 was used to give 10,000 points from which parameters were estimated. When performing model-choice between the suggested different scenarios 2,000,000 points were simulated and a tolerance of δ = 0.005 was used. We used the mode of the posterior distributions as a point estimate of the parameter. Credible intervals were calculated around the mode, following previous studies by Hamilton et al. [58] and Beaumont [53].

The model-choice studies were performed by first carrying out the simulation in parallel on a 24-node cluster, and then combining the simulated output, in order to shorten the simulation time. A program developed by Lopes and co-workers was used to simulate genetic data in an "Isolation with migration" model for any number of modern populations [31]. This software allows the use of STR's and single nucleotide polymorphism (SNP) data simultaneously. The regression step was performed using a script developed by Beaumont (makepd.r, ) under the free software environment R v2.5.0 [59]. The posterior density estimation from the adjusted sample of parameter values was carried out using the locfit function [60].

### Prior distributions of parameters

The same priors for the demographic parameters (current and ancestral effective sizes following a uniform distribution ranging from 1,000 to 100,000) were used for inferences based on the North-to-South speciation model (topology 1), and those based on South-to-North speciation model (topology 2, with *P. charlestoni *originating from a south-african ancestor) (Figure 2). The priors for the demographic parameters were chosen according to information available from the literature [34,35,61]. The priors for the splitting time estimates also followed a uniform prior, as indicated in Table 1. Mutation rates for each locus were treated as a nuisance parameter. Although we intended to differentiate between standard and slow mutation rates, it was not intended to infer their exact values. Therefore, a broad prior was used for the loci mutation rates to account for the uncertainty on the estimates. The variation in mutation rate between loci was accounted for by using a hierarchical Bayesian framework [62]. The mutation rates for each locus were drawn from a lognormal distribution (priors) with mean sampled from a normal distribution and the standard deviation being the absolute value sampled also from a normal distribution (hyper-priors) [53]. In order to cover the proposed limits of the mutation rate, we used a standard deviation hyperprior of 0.25 and chose for mtDNA a mean Standard mutation rate of 2.34 × 10-5 (log = -4.630784) and a Slow mutation rate of 5.4 × 10-6 (log = -5.267606) (Table 1). The use of hyper-parameters within ABC methods has been previously described [48,53,63].

**Table 1:** Divergence time and mutation rate hyper-parameter priors used for the ABC analyses.

**Divergence time parameters**		**Prior distribution**	
t_1_	First splitting time	Uniform (0, 2 My)	
t_2_	Second splitting time	t1 values + Uniform (0, 1.5 My)	
t_3_	Third splitting time	t2 values + Uniform (0, 1.5 My)	
t_4_	Fourth splitting time	t3 values + Uniform (0, 2 My)	
t_5_	Fifth splitting time	t4 values + Uniform (0, 5 My)	

**Marker Hyper-parameters**		**Mutation rate for mtDNA standard**	**slower**

ϑ_STRm_	mean of mutation rate for STR locus	Normal (-3.5, 0.25)	Normal (-3.5, 0.25)
ϑ_STRsd_	st. dev. of mutation rate for STR locus	Abs [Normal (0, 0.5)]	Abs [Normal (0, 0.5)]
ϑ_SNPm_	mean of mutation rate for mtDNA locus	Normal (-4.630784, 0.25)	Normal (-5.267606, 0.25)
ϑ_SNPsd_	st. dev. of mutation rate for mtDNA locus	Abs [Normal (0, 0.5)]	Abs [Normal (0, 0.5)]

### Choice of summary statistics

The summary statistics were chosen according to their success in previous ABC studies [49,53]. For mtDNA, 3 summary statistics were calculated for each sampled deme: number of haplotypes, k; number of segregating sites, S; and the average number of pairwise differences, π. For STR data, three within-deme summary statistics were calculated for each sampled deme: allele number, k; heterozygosity, H; and variance in allele length, Var(length). All this 6 statistics were computed for each of the six populations taken individually and for each of the fifteen pairs of populations pooled together. Hence, the Euclidian distances were computed from a total of 126 normalized summary statistics.

### Comparison of scenarios using approximate Bayesian computation

In order to test between previously proposed hypotheses (Figure 2), we considered four scenarios which differed in the population tree topology and in the prior distribution on the mutation rate: (1) sequential expansion from the Northern hemisphere and considering a normal prior distribution centred on a standard mutation rate (Pliocene-Pleistocene speciation); (2) North-to-South expansion, but considering a normal prior distribution centred on a slow mutation rate (Miocene speciation); (3) secondary colonization of the Northern hemisphere, with *P. charlestoni *originating from a *P. gilchristi*-like ancestor and considering a normal prior distribution centred on a standard mutation rate; and (4) secondary colonization of the Northern hemisphere but considering a normal prior distribution centred on a slow mutation rate. Therefore, an ABC framework was used to discriminate among our four different scenarios. This model-selection step was performed before estimating the final demographic historic parameters, which were done conditional to the most likely scenario. The prior probability for each scenario in all the comparisons were set to be equal (i.e. 1/2 for each two-scenario comparison). The posterior probability of each model was then estimated by performing the rejection-step followed by a logistic regression [53]. Priors for divergence times were made broad enough to consider both a Miocene and a Pleistocene speciation pattern (Table 1).

Beaumont [53] indicated that it is possible to sample the model indicator (i.e. {1, 2,..., m}) for "m" models (M1, M2,..., Mm) from a prior and treat this as a categorical random variable, X, in the ABC simulations. We can then apply a categorical regression to estimate P(X = x1|S = s'), where x = 1, 2,..., m is the indicator for model Mx and s' is the vector of the summary statistics that summarize our observed data. A scheme of weighting can also be used, with weights given by the Epanechnikov kernel, as done in a standard regression procedure. The regression-step was performed using Beaumont's R script calmod , which needs the VGAM package [64]. This procedure has been shown to substantially improve previous methods to select among different models using ABC [49,53].

## Results

### Classic distance-based methods

**Figure 3:**
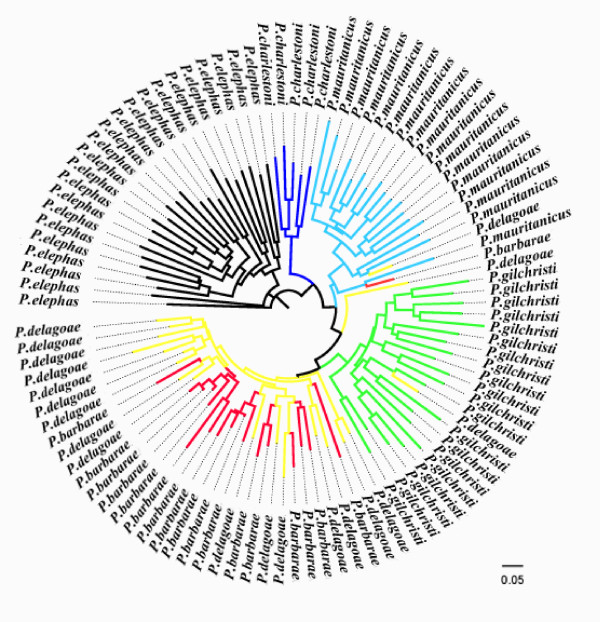
**Phylogenetic tree built from the individual-based distance matrix using the combined mtDNA-microsatellite dataset**. The Neighbor Joining algorithm under Cavalli-Sforza and Edwards distance measure agrees with placing *P. charlestoni *samples next to *P. elephas *samples. Species are coded by colors.

**Figure 4:**
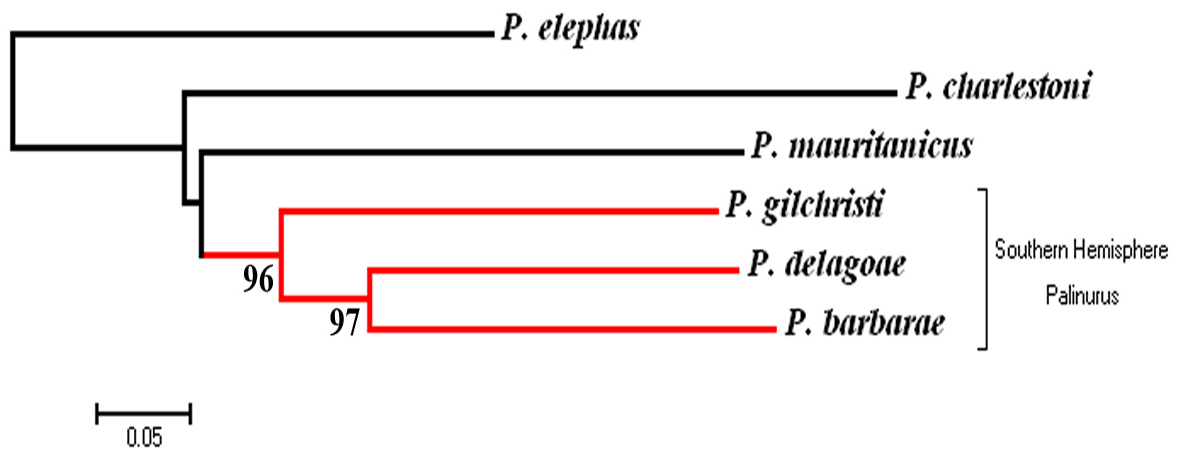
**Species tree obtained using the Neighbor Joining algorithm on the Cavalli-Sforza and Edwards distance matrix from the combined mtDNA-microsatellite dataset**. A well supported monophyletic southern hemisphere clade was obtained when dealing with populations instead of individuals. Only bootstrap support values over 70 are shown.

All microsatellite markers were polymorphic in every tested species with the exception of PE10 in *P. barbarae *(Additional file 1). A Mantel test (R = 0.626; P = 0.003) revealed a significant correlation between the genetic distance matrix obtained from mtDNA sequence data and that obtained from microsatellites (Additional file 2). Moreover, the correlation between the matrix of geographic distance and each genetic distance matrix was always significant although higher for the microsatellite dataset (R = 0.585; P = 0.001) than for the mtDNA dataset (R = 0.237; P = 0.033). The distance measure of Cavalli-Sforza and Edwards showed a southern hemisphere species clade and agrees with placing *P. charlestoni *samples next to *P. elephas *samples when phylogenetic trees are built using the individual-based matrices and the Neighbor Joining algorithm (Figure 3). When dealing with species instead of individuals, a well supported monophyletic southern hemisphere clade was obtained as well, even though phylogenetic relationships among northern hemisphere species were not completely resolved (Figure 4).

### ABC methods

The posterior distributions of model comparisons are presented in Table 2. The simulations obtained using distributions centered on the slow and standard mutation rate allowed us to check for which mutation rate has a higher posterior probability regardless topology (Table 2a). The tests for mutation rate of both topology models gave a better support for a standard mutation rate. This support is fairly strong, giving about 90% of probability of having a standard mutation rate. After testing for the mutation rate, the comparison of topologies assuming a particular mutation rate distribution allows us to check for which topology presents a higher posterior probability. The comparisons between the two different speciation models (Figure 2) considering the standard mutation rate strongly support the North-to-South speciation model (Table 2b). Therefore, the most likely scenario was a standard mutation rate and topology 1. According to the model comparison results, demographic parameters were estimated by conditioning the ABC runs to the most supported model (North-to-South speciation with a standard mutation rate).

**Table 2:** Posterior probabilities for different scenarios regarding the mutation rate (2a) and tree topology (2b) models

a)		
**Considered scenario**	**Mutation rate**	**Posterior probability**
Topology 1 (North-to-South)	Slow	0.10
	Standard	**0.90**
Topology 2 (South-to-North)	Slow	0.11
	Standard	**0.89**

b)		

**Considered scenario**	**Expansion scenario**	**Posterior probability**
Standard mutation rate	Topology 1	**0.88**
	Topology 2	0.12

Estimations of modern population effective sizes using both mitochondrial sequences and microsatellite markers pointed to about 50,000 individuals for populations of *P. barbarae*, *P. delagoae*, *P. gilchristi *and *P. elephas *and between 80,000 to 100,000 individuals for *P. mauritanicus *and *P. charlestoni *(Table 3). Estimates of effective size of the ancestor populations were not very informative given the large confidence intervals obtained (Additional file 3). The estimation of the second ancestor population though had a quite informative posterior distribution, showing a value of about 10,000 individuals. This ancestor population refers to the original population from the South African coast that later originated *P. gilchristi *and from which *P. barbarae *and *P. delagoae *originated from. This value corresponds to the effective population size, so it is not straightforward to infer the census population size from it. Nevertheless, these results seem to point out an expansion event of the referred south-African ancestral population.

**Table 3:** Estimates for mode and 95% credible intervals when the simulation is conditional to the North-to-South speciation with a standard mutation rate.

**Parameters**	**Description**	**Mode**	**0.95 C.I**.	
Ne1	*P. barbarae *population size	57.079	32.208	97.228
Ne2	*P. delagoae *population size	49.030	25.139	89.307
Ne3	*P. charlestoni *population size	49.752	26.376	92.307
Ne4	*P. gilchristi *population size	87.317	49.406	100.000
Ne5	*P. mauritanicus *population size	47.426	25.733	86.020
Ne6	*P. elephas *population size	97.604	63.970	100.000
NeA1	First ancestral population size	34.762	1.000	87.000
NeA2	Second ancestral population size	11.634	1.000	21.851
NeA3	Third ancestral population size	28.861	1.000	66.139
NeA4	Fourth ancestral population size	14.881	1.000	50.861
NeA5	Fifth ancestral population size	46.089	1.000	62.218
t1	First splitting time	89.209	15.602	213.021
t2	Second splitting time	200.220	80.858	358.436
t3	Third splitting time	552.555	280.528	886.589
t4	Fourth splitting time	975.198	532.753	1.474.347
t5	Fifth splitting time	1.765.377	952.895	2.625.863

In this ABC study, the estimates of demographic parameters which had a better support were the ones regarding splitting time. When conditioning for a North-to-South speciation pattern, all five splitting times showed a posterior distribution considerably different from the prior and quite tight around the mode (Table 3). Accordingly, the splitting time when both *P. elephas *and *P. mauritanicus *were originated was placed around 2 million years ago (mya), separation between *P. mauritanicus *and *P. charlestoni *lineages took place about 1 mya, colonization of South Africa would have taken place only 0.5 mya, and the appearance of both *P. delagoae *and *P. barbarae *would be placed at about 0.2 mya and 0.1 mya, respectively.

## Discussion

Our within-genus phylogenetic analyses using a new set of polymorphic nuclear loci and classic distance-based methods consistently support a North-to-South pattern of speciation in *Palinurus *with all the South African species forming a monophyletic clade (topology 1; Figures 2, 4). Moreover, the combination of nuclear and mtDNA markers under recently developed coalescent-based ABC methods has allowed us to test for the previously suggested hypothesis of *P. charlestoni *originating from a *P. gilchristi*-like ancestor [18]. It is known that when we are interested in the old events in a gene's history, small samples are sufficient, while if we are interested in recent events then larger sample sizes are critical [65]. Even though a larger sample size would allow us to better characterize the coalescent properties for each species (e.g. times to coalescence), and we were able to obtain only 5 *P. charlestoni *samples from Cape Verde Islands, our results show that a North-to-South speciation pattern occurring during the last 2 my is more consistent with the observed dataset.

A previous phylogeographic study using the COI region and standard rates for decapod crustaceans [33] found divergence times for *P. elephas *and *P. mauritanicus *to be much more recent [18]. Also, a previous study on the Achelata infraorder evolutionary relationships showed no genetic variation to be present among most *Palinurus *species when analyzed using sequence data from several nuclear genes [38]. These results already suggested that the speciation process within the genus *Palinurus *is fairly recent and therefore not related with the Middle Miocene closure of the Tethyan Seaway. In agreement with previous evidence, the coalescent simulations carried out in the present study indicate that the observed molecular dataset is not likely to result from a low mutation rate, while the standard mutation rate is supported regardless of the speciation hypothesis assumed. Most interestingly, divergence time estimates obtained for *Palinurus *species using standard rates agree with known glaciation-related processes occurring over the last 2 my [66].

The late Pliocene changes in the climate system, both in the Northern and the Southern Hemisphere, had a large impact on the evolution of many terrestrial organisms [67]. The gradual closure of the Panama Isthmus between 5 and 3 mya stopped the exchange of tropical Atlantic and Pacific water masses and the present day overall circulation pattern was established, with a strong influence of North Atlantic Deep Water on the global circulation and intensification of the Benguela Current upwelling system [66,68]. The Matuyama diatom maximum (~2 mya) marked the transition to a cold mode of trade-wind controlled upwelling along the Southwest African coast with enhanced advection of sub-Antarctic water masses [69]. Marlow et al. [70] have shown that the Benguela Current upwelling system became pronounced at 2.1 to 1.9 mya and intensified during the period leading to the onset of the 100 ky glacial cycles at about 0.6 mya. Consequently, the intensification of Benguela Current upwelling had a direct regional influence by cooling the marine waters of southern Africa, with average sea surface temperature shifting from about 26°C in the mid-Pliocene (3.5 mya) to approximately 11-17°C in the present [24,70]. This could have facilitated the colonization of southern Africa from the North, since temperate-water *Palinurus *species are generally found between 11-16°C [9,18]. Interestingly, ABC divergence time estimates for the separation of *P. charlestoni *and *P. gilchristi *lineages would correspond to about 550 ky ago, after Southwest African waters became suitable for *Palinurus *species.

Even though the results obtained in previous studies provide comforting genealogical support to the classical view of species as qualitatively distinct taxa [18,71], the reconstruction provided by mtDNA is not representative of most of the genome and may bias perceptions of evolutionary diversification [72]. The demographic context of differentiation is not taken into account in most mtDNA studies because single loci offer low precision on estimates of historical population size [28] and because the relatively shallow coalescent time for this molecule limits the temporal window for demographic inferences [73]. For a given divergence time, historical population size is a key factor determining whether a species is polymorphic at most loci and whether genes are expected to accurately trace the species phylogeny. It is widely recognized that large populations undergoing rapid speciation, such as in some marine species, could create intermingled genealogical tracings containing very little phylogenetic information among species divergence [74].

Some doubts had been previously raised on the phylogenetic relationships of the extant species of the spiny lobster genus *Palinurus*, since they show very few morphological differences, with overlaps between the Indian and Atlantic Ocean taxa [75]. In fact, most *Palinurus *species cannot be discriminated using sequences for several nuclear coding genes and *P. delagoae *and *P. gilchristi *have been found to share 16S haplotypes [18,33]. Even though nesting of some individuals within a different species may be caused by some microsatellites being identical in allele-size without being identical by descent (homoplasy), the results obtained from individual-based analyses using mtDNA and microsatellite markers indicate that monophyly patterns in *P. delagoae *and *P. barbarae *are not well supported (Figure 3). The coalescent theory has shown that polyphyletic gene lineages can persist in species long enough after divergence [72,76]. With an ancestral population effective size of about 20,000 [34] and a generation time of 4-10 years [36], an average of 160-400 ky would be needed for gene lineages to get fixed in *Palinurus *species. This would be in agreement with divergence time estimates obtained from the combined dataset, since individual-based analyses show that incomplete lineage sorting is most pronounced in the most recently evolved species pair, *P. delagoae *and *P. barbarae *(divergence time: ~89 kyr).

## Conclusion

The combination of markers from both nuclear and mitochondrial genomes under an ABC-coalescent framework has proven to be effective for testing among alternative evolutionary hypotheses in *Palinurus *and highlights the importance of using multiple markers when dealing with closely-related species. The *Palinurus *speciation pattern is a typical example of a series of rapid speciation events occurring within a group, with very short branches separating different species. Therefore, it has been shown that molecular tools can provide new insights into the mechanisms, pace and geography of marine speciation. Indeed, recent genetic evidence suggests that many species groups are relatively new, originating after the onset of the Pleistocene, during the last two million years [5,77]. These recent speciation events provide a great opportunity to analyze the speciation process in marine taxa, since footprints of species formation are most likely to be identified when comparing recently diverged species, the initial differentiation of which can be correlated with the different proposed speciation processes.

## Authors' contributions

FP designed the study, obtained the majority of the samples, carried out the laboratory work, did most of the analyses and prepared the manuscript. JL and MB provided their knowledge on ABC methods and allowed for access to the popABC software and helped with the coalescent-simulation work. PA, EM and MP provided guidance and supervision during the research, contributed to the preparation of the manuscript and helped with tissue sample acquisition. All authors read and approved the final version.

## Supplementary Material

Additional file 1 **Gene diversity values for each locus**. The table provided represent gene diversity values in every microsatellite locus for each *Palinurus *species. Click here for file

Additional file 2 **Pairwise distance estimates for geographic (km) and genetic data (Fst) using different markers**. The tables provided include the pairwise geographic distance matrix (km) and genetic distance matrices obtained using mitochondrial DNA and microsatellite markers. Click here for file

Additional file 3 **Posterior probabilities obtained for different parameters using ABC methods**. The figures show the posterior (blue) and prior (black) distribution for the estimation of parameter values. Click here for file
