# Supplementary material for: Rapid radiation in spiny lobsters (Palinurus spp) as revealed by classic and ABC methods using mtDNA and microsatellite data
Source: BMC Evol Biol. 2009 Nov 9;9:263. doi: 10.1186/1471-2148-9-263 (PMC2777881; doi:10.1186/1471-2148-9-263)
Supplement: Additional file 1 — Gene diversity values for each locus. The table provided represent gene diversity values in every microsatellite locus for each Palinurus species. [file 1471-2148-9-263-S1.pdf]

	P. elephas	*P. mauritanicus*	*P. charlestoni*	*P. gilchristi*	*P. delagoae*	*P. barbarae*
**Pael-10**	0.351	0.166	0.533	0.050	0.097	-
**Pael-11**	0.634	0.494	0.378	0.789	0.645	0.733
**Pael-12**	0.937	0.882	0.933	0.867	0.883	0.870
**Pael-14**	0.926	0.757	0.911	0.918	0.869	0.902
**Pael-20**	0.865	0.954	0.956	0.959	0.883	0.918
**Pael-21**	0.918	0.765	0.711	0.722	0.774	0.743
**Pael-22**	0.520	0.568	0.714	0.603	0.520	0.542
**Pael-28**	0.821	0.770	0.778	0.701	0.500	0.703
**Pael-31**	0.899	0.803	0.929	0.868	0.864	0.517
**Pael-44**	0.964	0.938	0.933	0.969	0.956	0.960
**Pael-48**	0.794	0.570	0.889	0.879	0.881	0.694
**Pael-49**	0.882	0.775	0.511	0.776	0.709	0.661
**Pael-53**	0.782	0.405	0.733	0.765	0.323	0.223

All markers were shown polymorphic in every tested species with the exception of PE10 in *P. barbarae*.
