# Supplementary material for: Rapid radiation in spiny lobsters (Palinurus spp) as revealed by classic and ABC methods using mtDNA and microsatellite data
Source: BMC Evol Biol. 2009 Nov 9;9:263. doi: 10.1186/1471-2148-9-263 (PMC2777881; doi:10.1186/1471-2148-9-263)
Supplement: Additional file 2 — Pairwise distance estimates for geographic (km) and genetic data (Fst) using different markers. The tables provided include the pairwise geographic distance matrix (km) and genetic distance matrices obtained using mitochondrial DNA and microsatellite markers. [file 1471-2148-9-263-S2.pdf]

	Geographic distance (Km)				
	P.elephas	***P.mauritanicus***	***P.charlestoni***	***P.gilchristi***	***P.delagoae***
P.mauritanicus	1098				
P.charlestoni	2468	1370			
P.gilchristi	10338	9240	7870		
P.delagoae	11833	10735	9365	1495	
P.barbarae	12538	11440	10070	2200	705

	GTR Genetic distance (mtDNA data)				
	P.elephas	***P.mauritanicus***	***P.charlestoni***	***P.gilchristi***	***P.delagoae***
P.mauritanicus	8.13				
P.charlestoni	7.54	5.00			
P.gilchristi	6.70	4.69	3.01		
P.delagoae	7.86	6.10	3.56	3.71	
P.barbarae	8.24	6.05	3.77	4.26	3.32

	Locus:	All msat loci			
	P.elephas	***P.mauritanicus***	***P.charlestoni***	***P.gilchristi***	***P.delagoae***
P.mauritanicus	0.164				
P.charlestoni	0.1637	0.1944			
P.gilchristi	0.1467	0.1303	0.1285		
P.delagoae	0.1972	0.1893	0.1383	0.0881	
P.barbarae	0.2164	0.1957	0.1897	0.095	0.0247

	Locus:	PE10			
	P.elephas	***P.mauritanicus***	***P.charlestoni***	***P.gilchristi***	***P.delagoae***
P.mauritanicus	0.6445				
P.charlestoni	0.5891	0.4994			
P.gilchristi	0.6679	-0.0021	0.7024		
P.delagoae	0.6447	-0.0042	0.64	-0.0169	
P.barbarae	0.6756	0.0455	0.7713	-0.0027	-0.0054

	Locus:	PE11			
	P.elephas	***P.mauritanicus***	***P.charlestoni***	***P.gilchristi***	***P.delagoae***
P.mauritanicus	0.2408				
P.charlestoni	0.4197	0.4716			
P.gilchristi	0.2904	0.2706	0.1878		
P.delagoae	0.3624	0.3719	-0.0031	0.132	
P.barbarae	0.3397	0.3104	0.1298	-0.0323	0.0833

	Locus:	PE12			
	P.elephas	***P.mauritanicus***	***P.charlestoni***	***P.gilchristi***	***P.delagoae***
P.mauritanicus	0.0173				
P.charlestoni	-0.0097	0.008			
P.gilchristi	0.0235	0.0515	-0.0054		
P.delagoae	0.0159	0.0204	0.0095	0.0045	
P.barbarae	0.0236	0.0309	-0.0159	0.003	0.002

	Locus:	PE14			
	P.elephas	***P.mauritanicus***	***P.charlestoni***	***P.gilchristi***	***P.delagoae***
P.mauritanicus	0.0348				
P.charlestoni	0.0356	0.1201			
P.gilchristi	-0.0005	0.0641	0.0195		
P.delagoae	0.0208	0.0526	0.045	0.0089	
P.barbarae	0.0326	0.0901	0.0224	0.0104	0.0281

	Locus:	PE20			
	P.elephas	***P.mauritanicus***	***P.charlestoni***	***P.gilchristi***	***P.delagoae***
P.mauritanicus	0.0353				
P.charlestoni	0.0126	-0.0179			
P.gilchristi	0.0464	-0.0041	-0.0087		
P.delagoae	0.0694	0.0079	-0.0121	0.0166	
P.barbarae	0.0729	0.0083	-0.0055	0.0049	-0.0082

	Locus:	PE21			
	P.elephas	***P.mauritanicus***	***P.charlestoni***	***P.gilchristi***	***P.delagoae***
P.mauritanicus	0.1273				
P.charlestoni	0.1283	0.085			
P.gilchristi	0.1384	0.2025	0.2338		
P.delagoae	0.1188	0.1276	0.0344	0.0772	
P.barbarae	0.133	0.1535	0.061	0.1263	0.0032

	Locus:	PE22			
	P.elephas	***P.mauritanicus***	***P.charlestoni***	***P.gilchristi***	***P.delagoae***
P.mauritanicus	0.0636				
P.charlestoni	0.2658	0.2297			
P.gilchristi	0.1791	0.141	0.1778		
P.delagoae	0.3403	0.3425	0.2482	0.0756	
P.barbarae	0.3494	0.3384	0.2461	0.0914	-0.0299

	Locus:	PE28			
	P.elephas	***P.mauritanicus***	***P.charlestoni***	***P.gilchristi***	***P.delagoae***
P.mauritanicus	0.1242				
P.charlestoni	0.0932	0.0652			
P.gilchristi	0.1109	0.2203	0.1047		
P.delagoae	0.2227	0.3091	0.0811	0.2066	
P.barbarae	0.1077	0.2115	0.0294	0.1392	0.0569

	Locus:	PE31			
	P.elephas	***P.mauritanicus***	***P.charlestoni***	***P.gilchristi***	***P.delagoae***
P.mauritanicus	0.1063				
P.charlestoni	0.0812	0.0558			
P.gilchristi	0.101	0.0549	0.0694		
P.delagoae	0.0882	0.0473	0.0081	0.0098	
P.barbarae	0.2342	0.1273	0.2546	0.1263	0.0209

	Locus:	PE44			
	P.elephas	***P.mauritanicus***	***P.charlestoni***	***P.gilchristi***	***P.delagoae***
P.mauritanicus	0.0218				
P.charlestoni	0.0218	0.0375			
P.gilchristi	0.0151	0.0123	0.0272		
P.delagoae	0.0101	0.0111	0.004	-0.009	
P.barbarae	0.0235	0.0231	-0.0021	0.0208	0.0087

	Locus:	PE48			
	P.elephas	***P.mauritanicus***	***P.charlestoni***	***P.gilchristi***	***P.delagoae***
P.mauritanicus	0.2901				
P.charlestoni	0.1974	0.3092			
P.gilchristi	0.0749	0.201	0.0826		
P.delagoae	0.1078	0.2448	0.0108	0.0129	
P.barbarae	0.1738	0.3395	0.2055	0.0722	0.0732

	Locus:	PE49			
	P.elephas	***P.mauritanicus***	***P.charlestoni***	***P.gilchristi***	***P.delagoae***
P.mauritanicus	0.1078				
P.charlestoni	0.2374	0.3207			
P.gilchristi	0.1077	0.1281	0.3066		
P.delagoae	0.1366	0.1315	0.3323	0.2054	
P.barbarae	0.1657	0.0556	0.394	0.2094	0.0378

	Locus:	PE53			
	P.elephas	***P.mauritanicus***	***P.charlestoni***	***P.gilchristi***	***P.delagoae***
P.mauritanicus	0.2534				
P.charlestoni	0.0846	0.4485			
P.gilchristi	0.1474	0.2416	0.0363		
P.delagoae	0.3293	0.5802	0.5159	0.3109	
P.barbarae	0.3632	0.6285	0.6118	0.357	-0.0184
