# Supplementary material for: Rapid radiation in spiny lobsters (Palinurus spp) as revealed by classic and ABC methods using mtDNA and microsatellite data
Source: BMC Evol Biol. 2009 Nov 9;9:263. doi: 10.1186/1471-2148-9-263 (PMC2777881; doi:10.1186/1471-2148-9-263)
Supplement: Additional file 3 — Posterior probabilities obtained for different parameters using ABC methods. The figures show the posterior (blue) and prior (black) distribution for the estimation of parameter values. [file 1471-2148-9-263-S3.pdf]

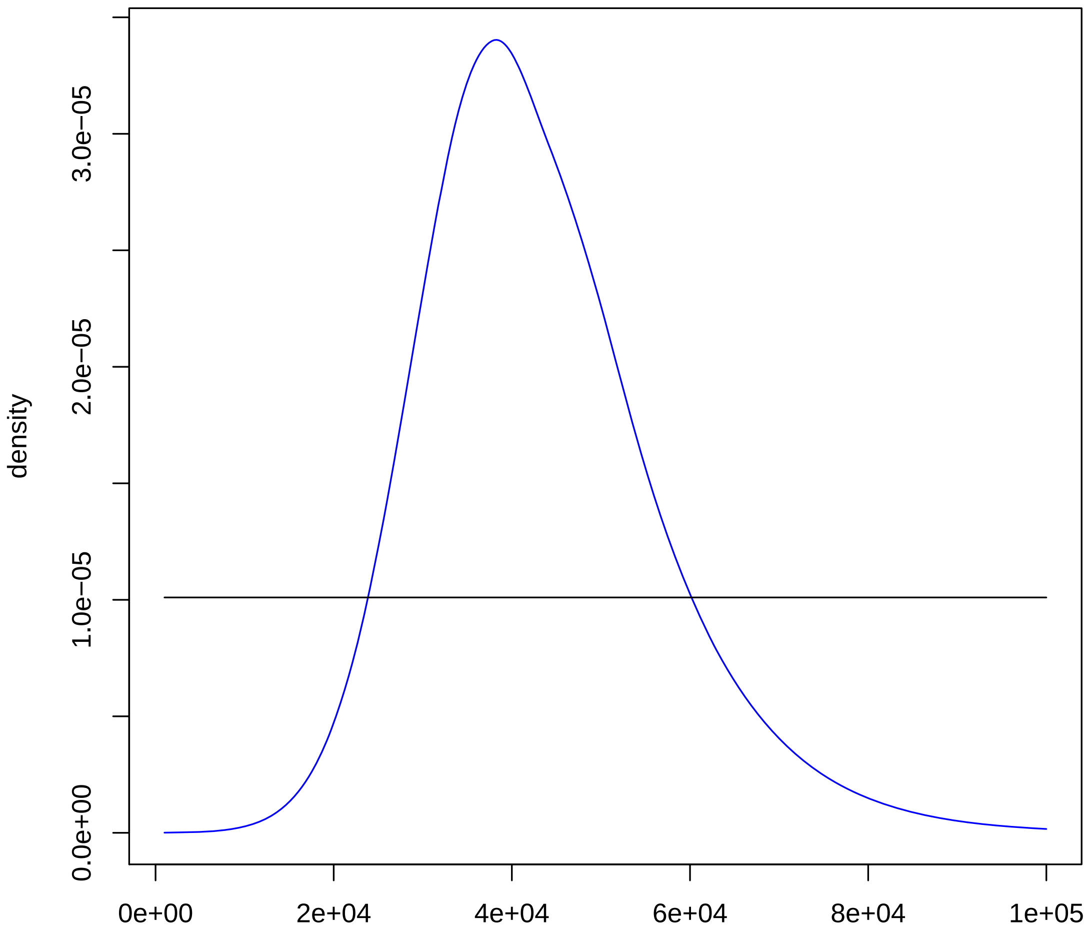

Fig. 1 – Posterior (blue) and prior (black) distribution for the estimation of *P. barbarae* population size (Ne_1).

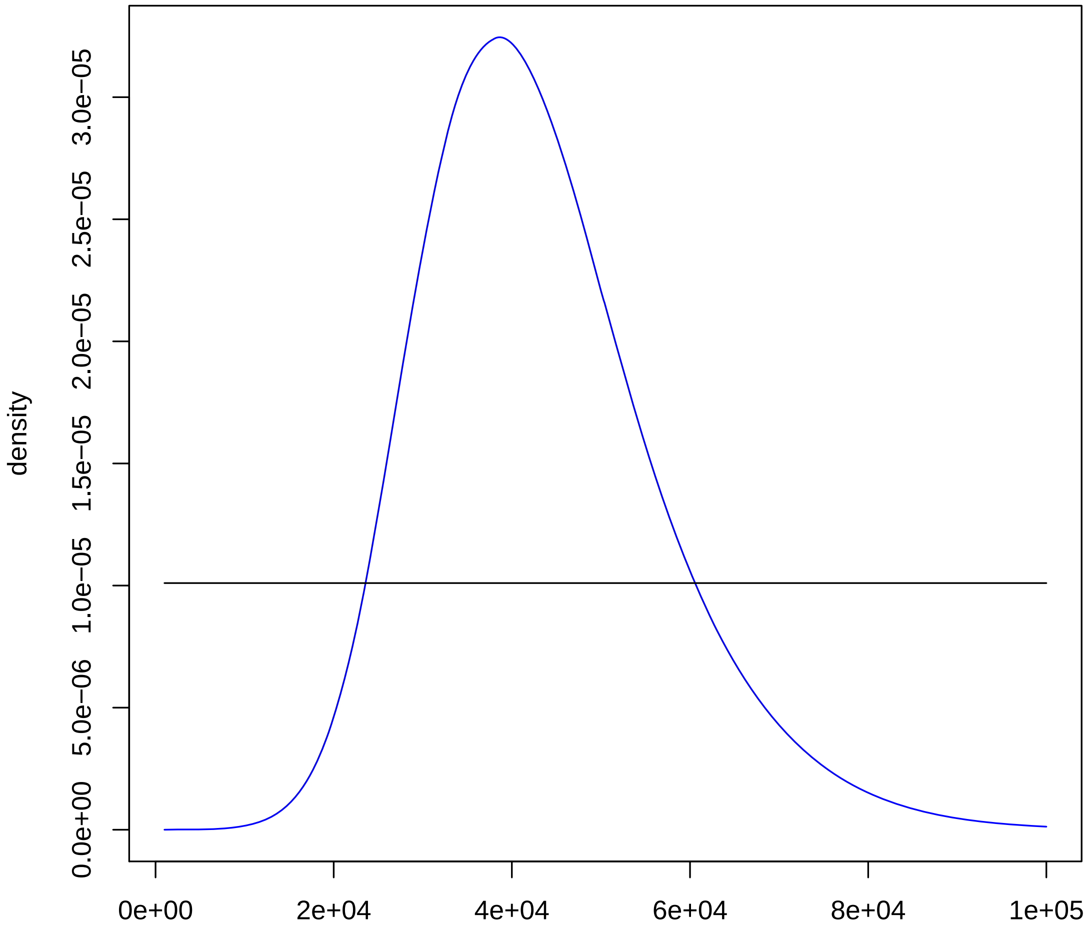

Fig. 2 – Posterior (blue) and prior (black) distribution for the estimation of *P. delagoae* population size (Ne_2).

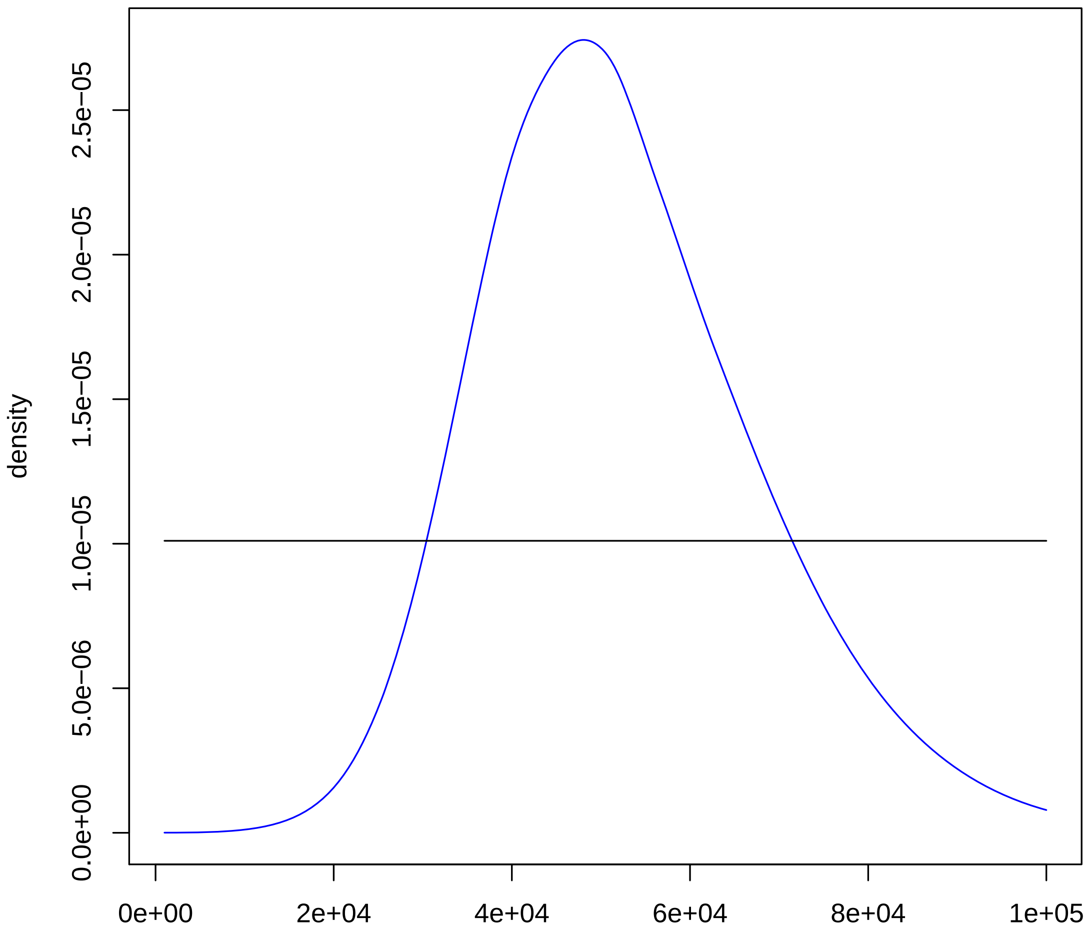

Fig. 3 – Posterior (blue) and prior (black) distribution for the estimation of *P. charlestoni* population size (Ne_3).

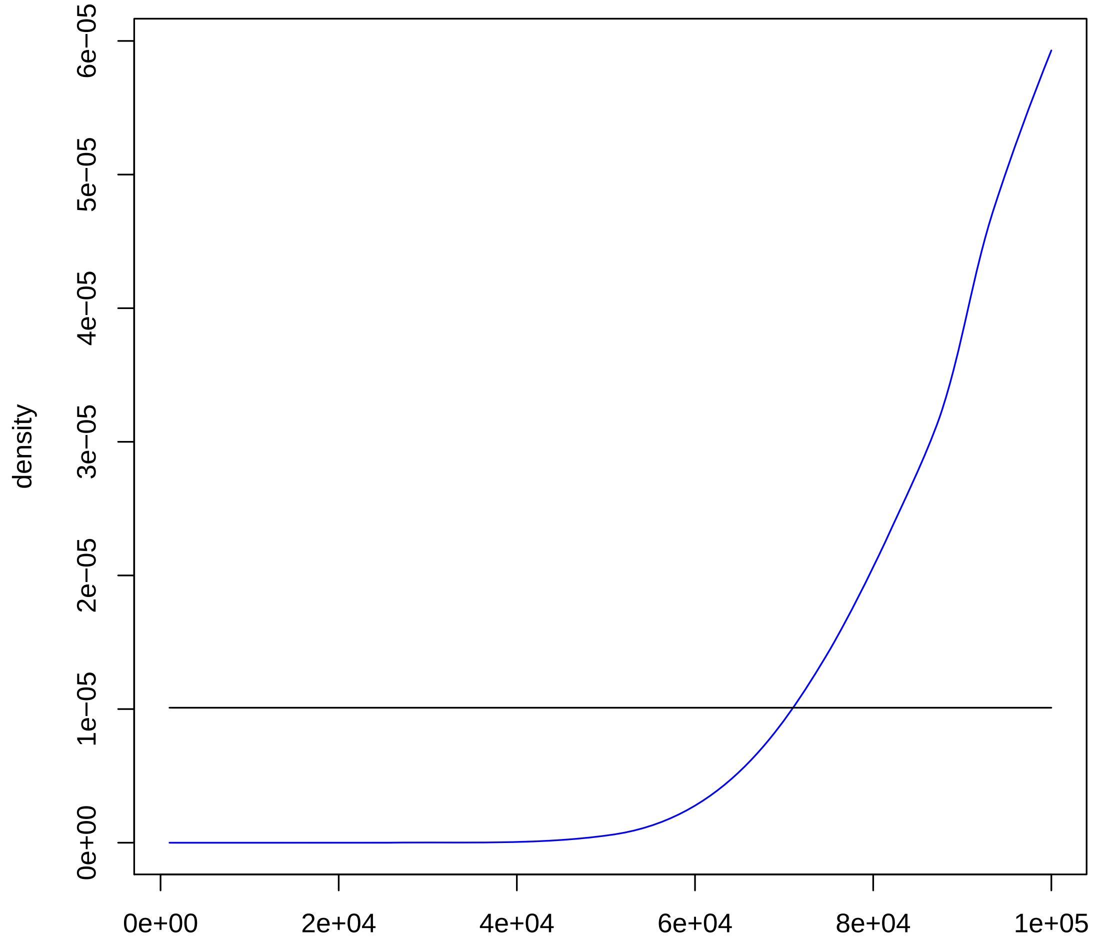

Fig. 4 – Posterior (blue) and prior (black) distribution for the estimation of *P. guilchristi* population size (Ne_4).

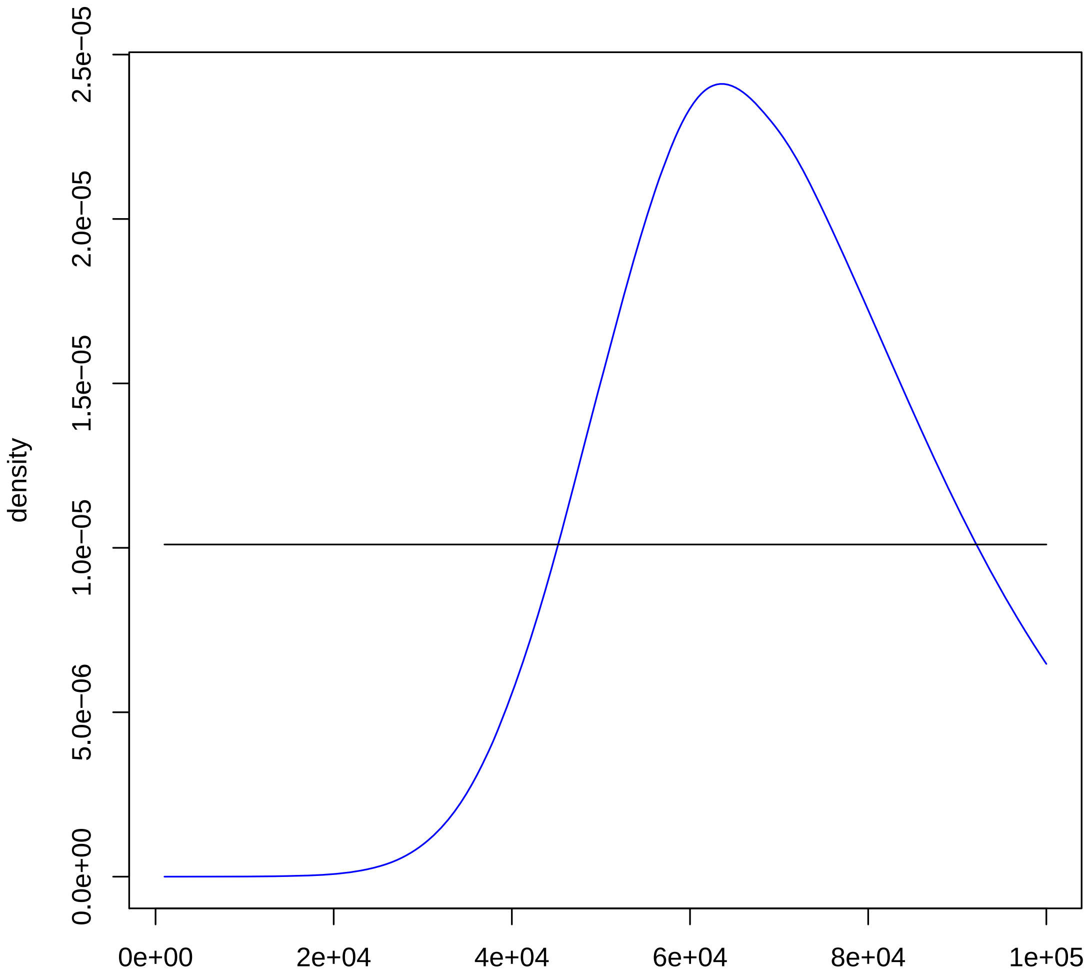

Fig. 5 – Posterior (blue) and prior (black) distribution for the estimation of *P. mauritanicus* population size (Ne_5).

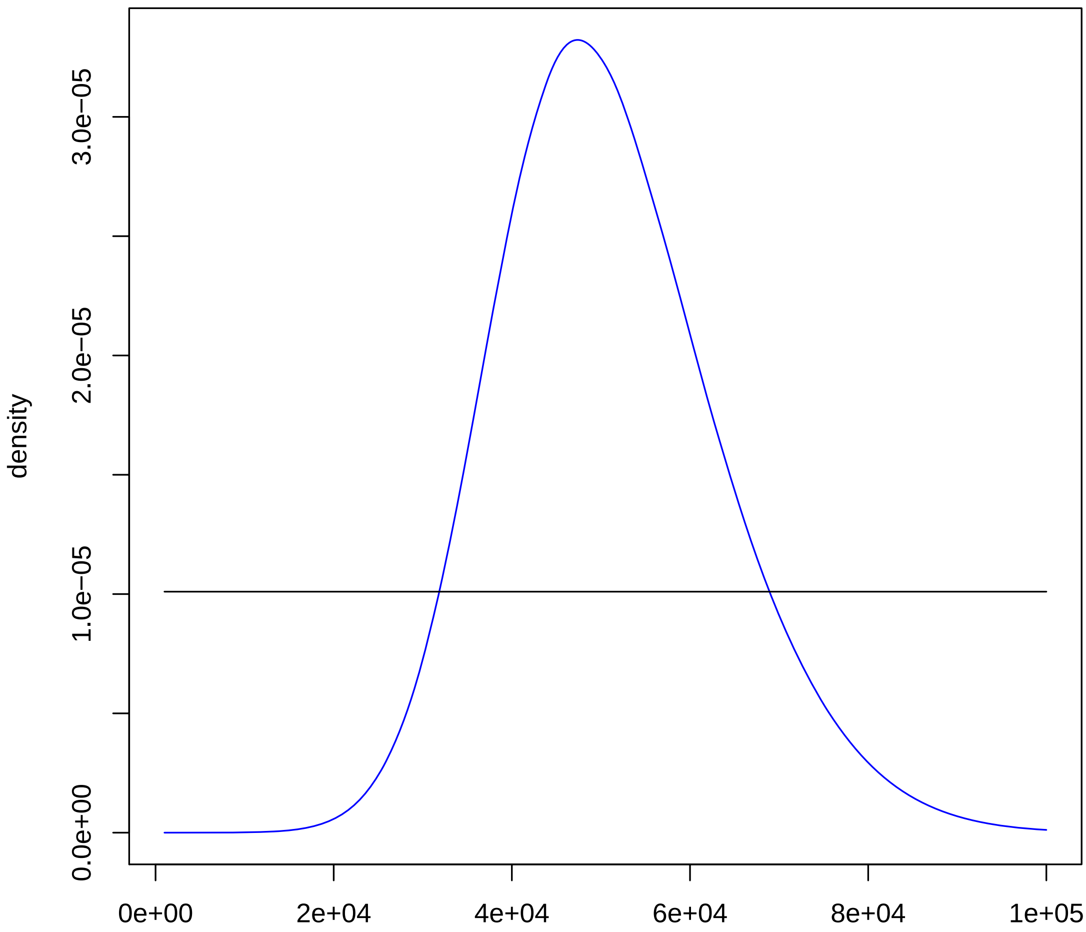

Fig. 6 – Posterior (blue) and prior (black) distribution for the estimation of *P. elephas* population size (Ne_6).

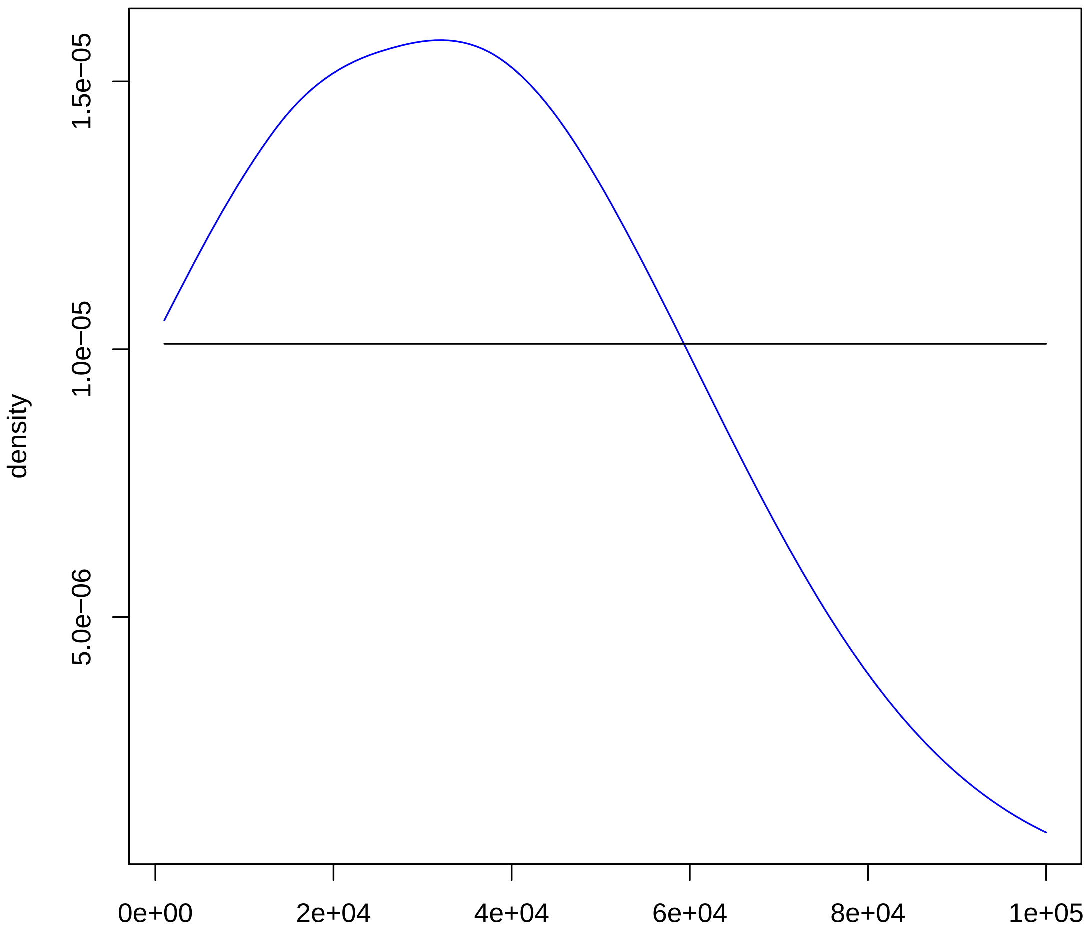

Fig. 7 – Posterior (blue) and prior (black) distribution for the estimation of the first ancestor population size (Ne_{A1}).

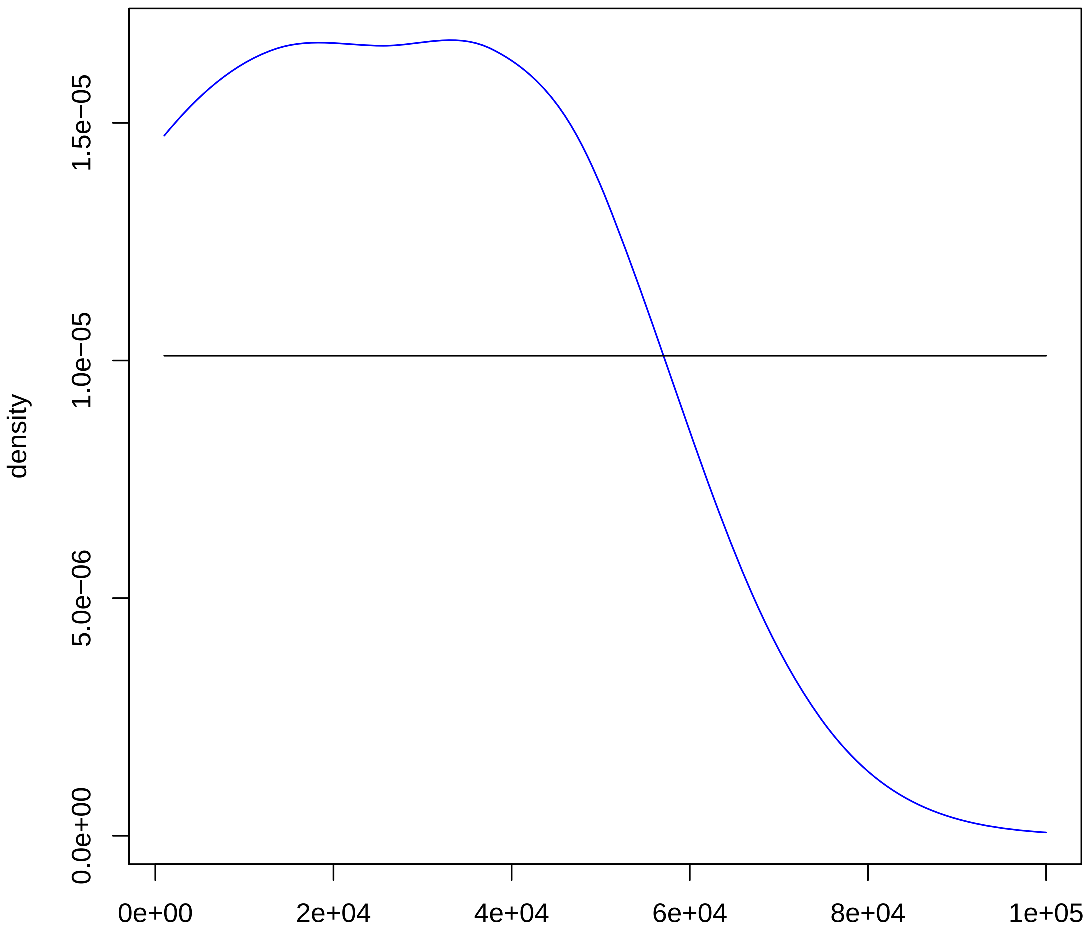

Fig. 8 – Posterior (blue) and prior (black) distribution for the estimation of the second ancestor population size (Ne_{A2}).

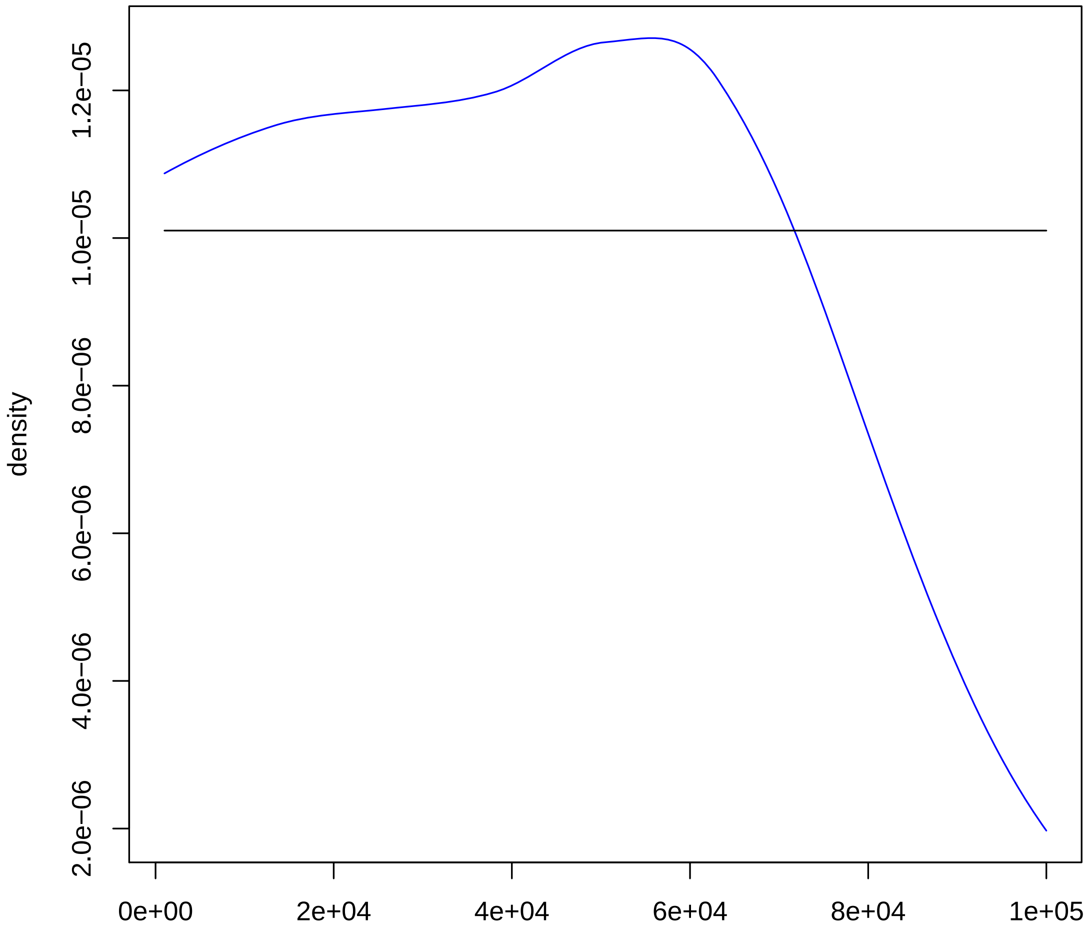

Fig. 9 – Posterior (blue) and prior (black) distribution for the estimation of the third ancestor population size (Ne_{A3}).

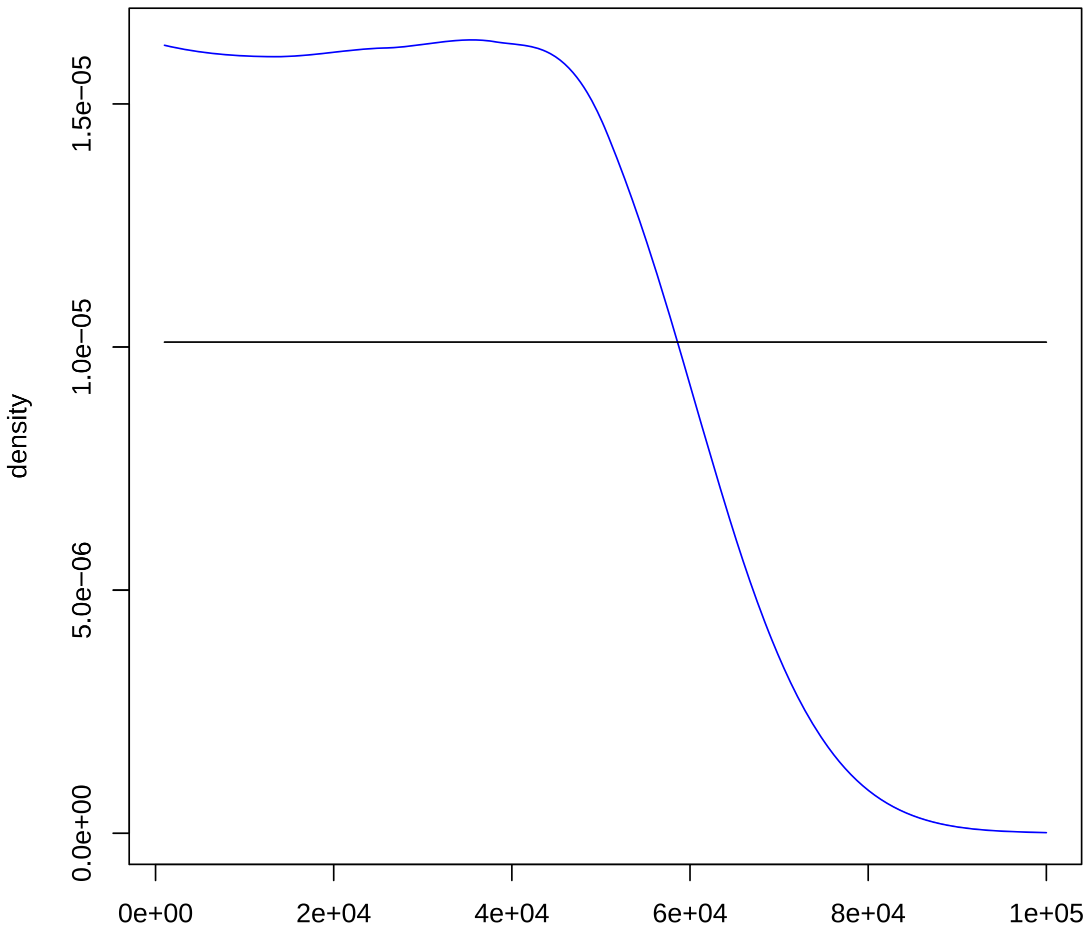

Fig. 10 – Posterior (blue) and prior (black) distribution for the estimation of the forth ancestor population size (Ne_{A4}).

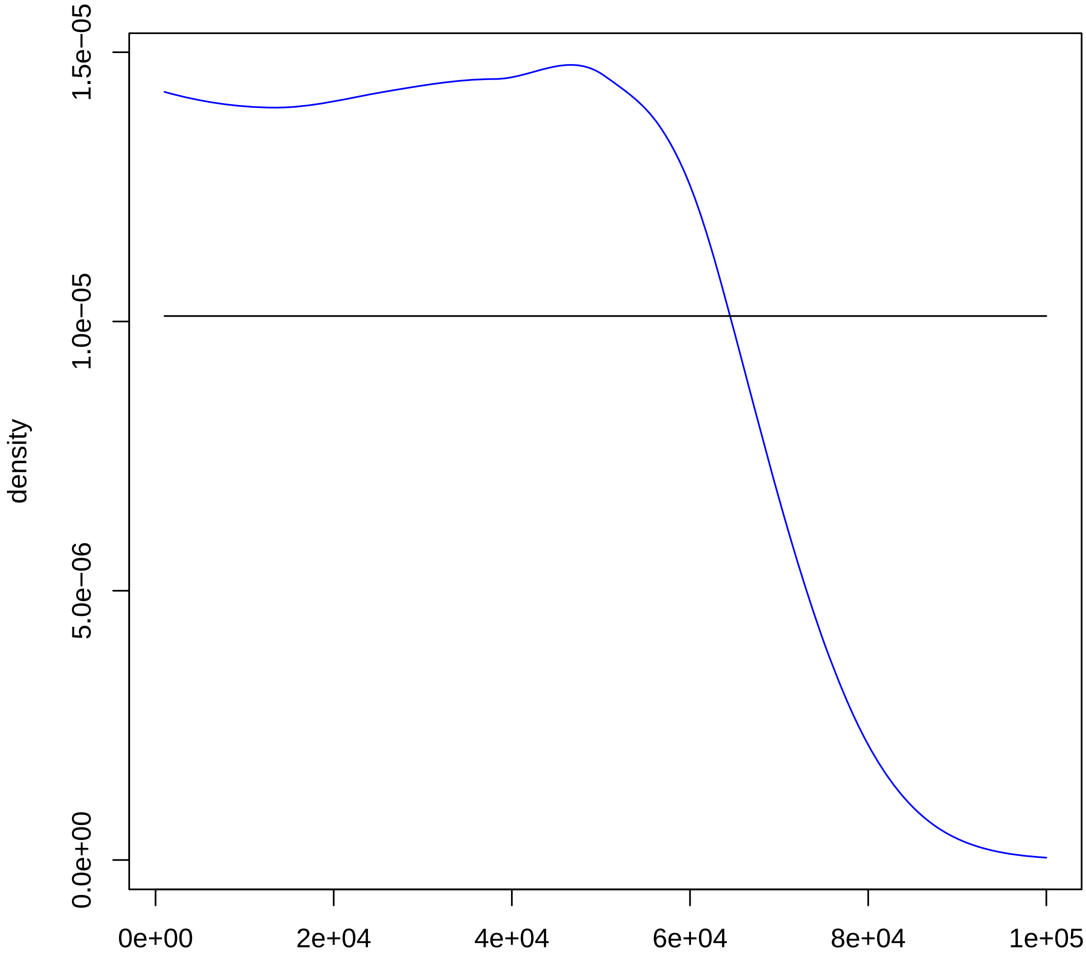

Fig. 11 – Posterior (blue) and prior (black) distribution for the estimation of the fifth ancestor population size (Ne_{A5}).

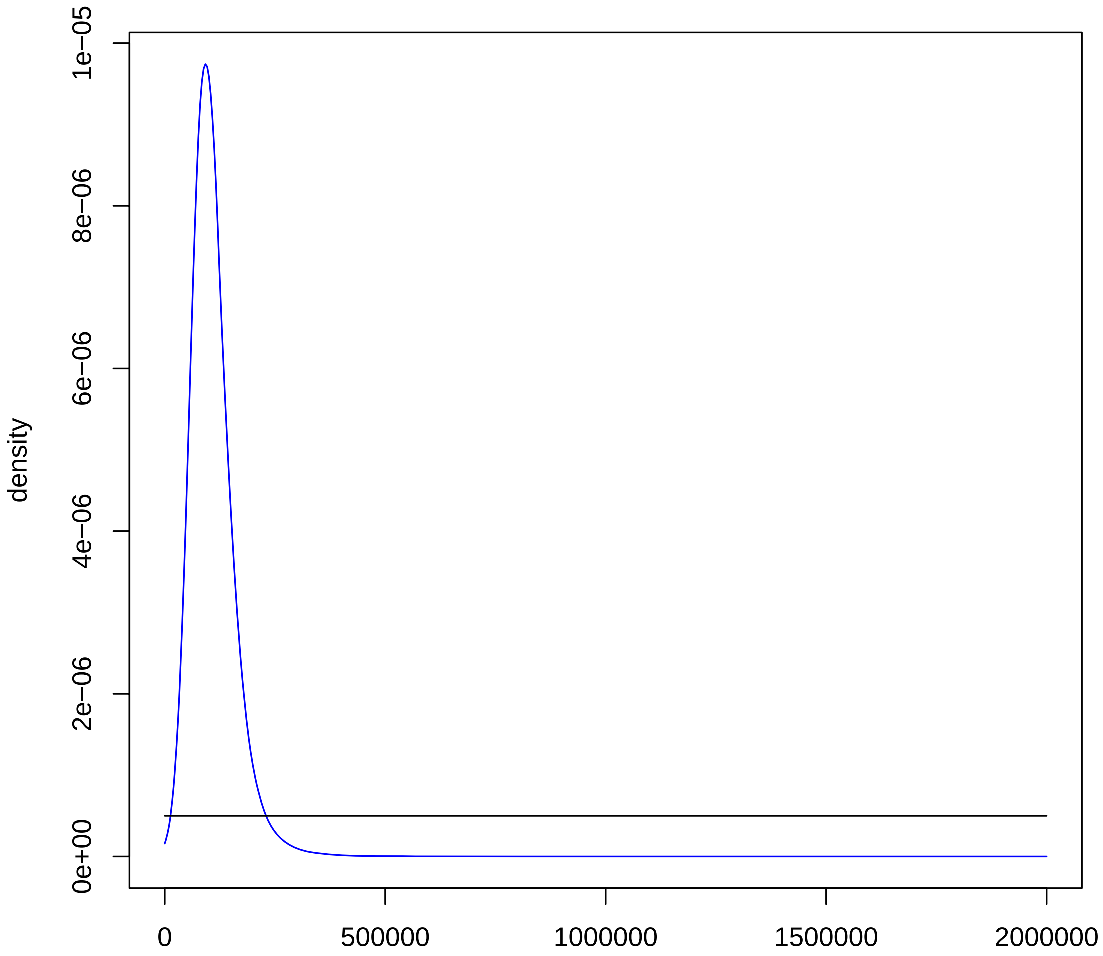

Fig. 12 – Posterior (blue) and prior (black) distribution for the estimation of first splitting event (t_1).

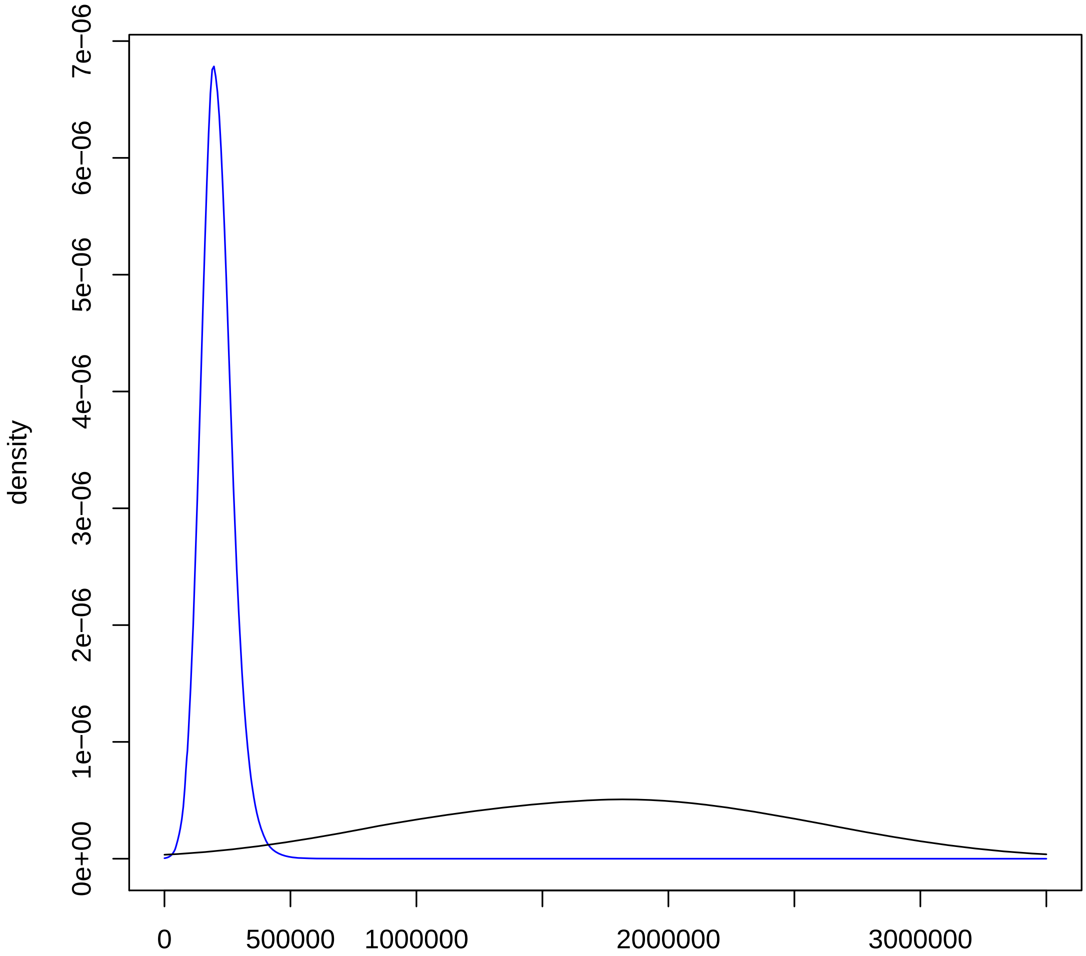

Fig. 13 – Posterior (blue) and prior (black) distribution for the estimation of second splitting event (t_2).

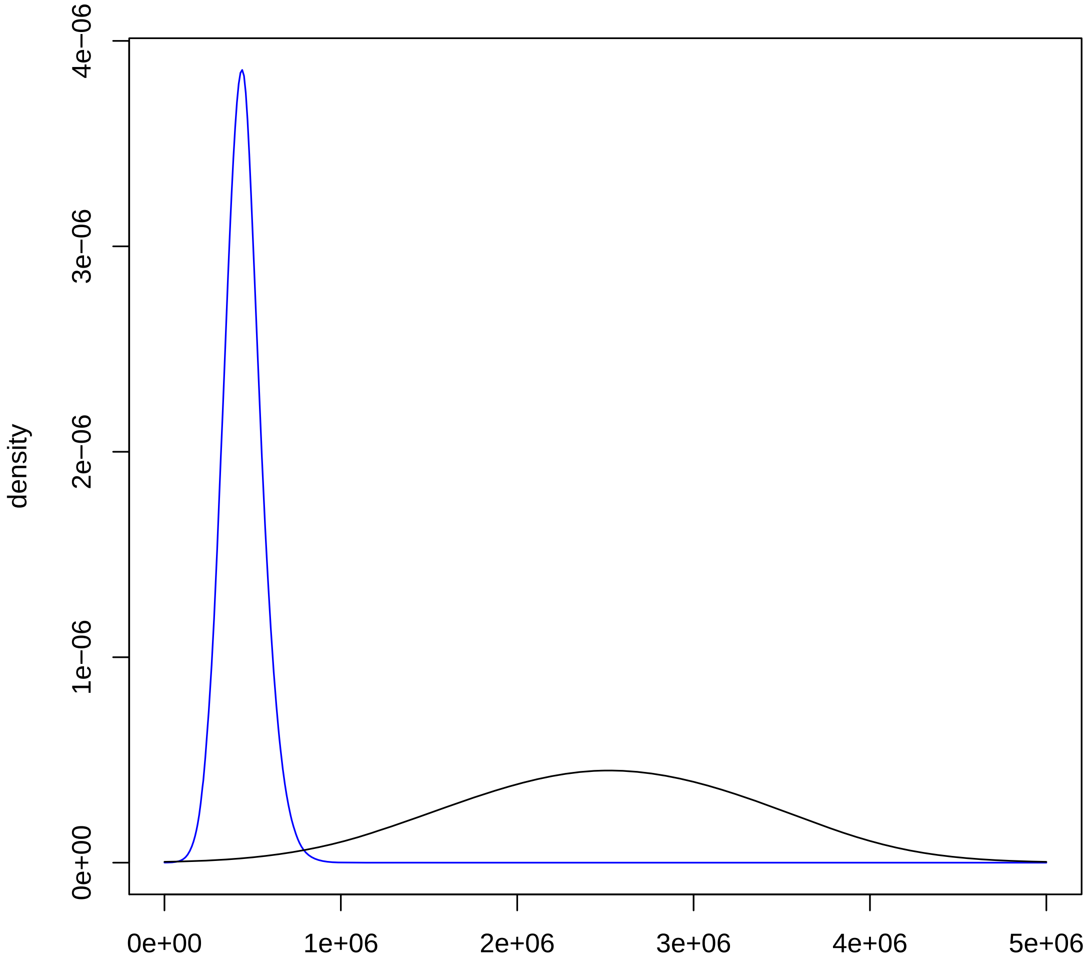

Fig. 14 – Posterior (blue) and prior (black) distribution for the estimation of third splitting event (t_3).

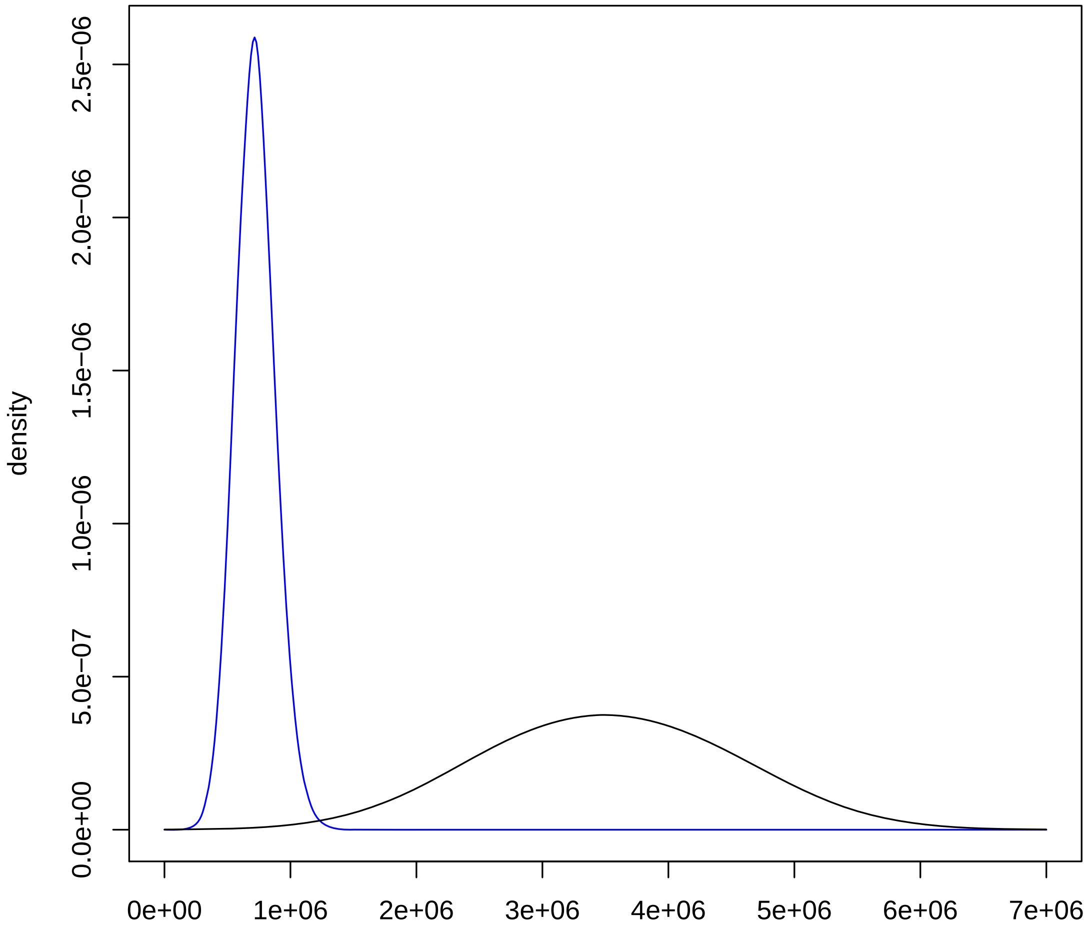

Fig. 14 – Posterior (blue) and prior (black) distribution for the estimation of forth splitting event (t_4).

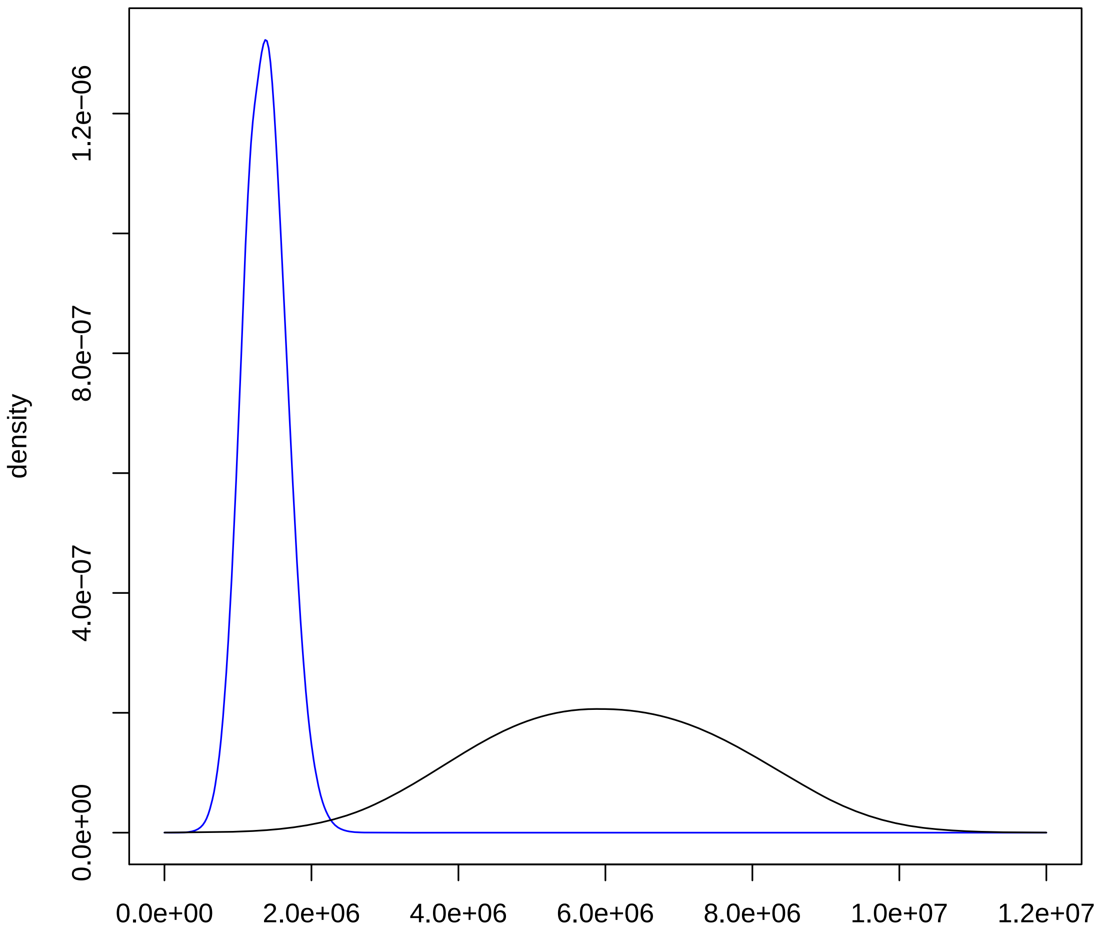

Fig. 15 – Posterior (blue) and prior (black) distribution for the estimation of fifth splitting event (t_5).
